# Swiprosin-1/EFhd2 – another piece in the puzzle of tauopathy?

**DOI:** 10.18632/aging.101431

**Published:** 2018-04-25

**Authors:** Martin Regensburger, Dirk Mielenz, Beate Winner

**Affiliations:** 1 Department of Stem Cell Biology, Friedrich-Alexander Universität Erlangen-Nürnberg (FAU), 91054 Erlangen, Germany; 2 Department of Neurology, FAU, 91054 Erlangen, Germany; 3 Department of Molecular Immunology, FAU, 91054 Erlangen, Germany

**Keywords:** Swiprosin-1, EFhd2, adult neurogenesis, neurodegeneration

The loss of neurons is an inevitable aging process. This may be aggravated by neurodegenerative diseases such as Alzheimer’s disease (AD). In addition to the death of mature neurons, adult neurogenesis declines with aging. The dentate gyrus (DG) and the subventricular zone (in mice) are the two neurogenic regions of the adult mammalian brain. Adult hippocampal neurogenesis comprises self-renewal of neural stem cells, proliferation of neural precursors and neuroblasts (week 0-1), differentiation and dendrite formation (week 1-4), synapse formation (~week 3-4) and integration into the granule cell layer (week 3-6) [1]. Dendrite and synapse formation depend upon differentiation signals, such as synaptic input or soluble factors, and require complex orchestration of the neuron’s actin and microtubule networks.

The reversible physiologic phosphorylation of the microtubule associated protein tau (TAU) modulates microtubule dynamics. On the contrary, intracellular neurofibrillary tangles consisting of insoluble, hyper-phosphorylated microtubule associated protein tau (p-TAU) are a neuropathological hallmark of AD. Accumulation of p-TAU occurs in other neurodegenerative diseases, including frontotemporal dementia and progressive supranuclear palsy. A causal role of TAU in these diseases is likely, given that mutations in *MAPT*, the gene encoding TAU, cause an inherited form of frontotemporal dementia and parkinsonism, and that the H2 haplotype of *MAPT* increases the risk of Parkinson’s disease [2].

EFHD2 (also termed Swiprosin-1) has two EF hands and a C-terminal coiled-coil domain. Upon Ca^2+^ binding, EFHD2 dimerizes and bundles F-Actin [3]. Phosphorylation of EFHD2 at S138 modulates lamellipodia dynamics and phosphorylation of EFHD2 at S74 by CDK5 modulates Ca^2+^ binding [3]. EFHD2 was identified in a complex with p-TAU in the brain of a transgenic mouse model for tauopathy (tauP301Ltg) as well as in old and diseased human brains [4].

We have previously shown that EFHD2 is highly expressed in the dendritic and axonal compartments in cortical, hippocampal and thalamic neurons throughout development and in the adult [5]. EFHD2 negatively regulated kinesin-dependent microtubule gliding in a cell-free assay, microtubule-dependent vesicle transport [5], and spine/ dendrite formation in primary mouse neurons [6]. This indicated that EFHD2 regulates formation and maintenance of synapses. *Efhd2* knockout mice show reduced cortical volumes but otherwise no gross abnormalities of brain development and anatomy [2,6]. No monogenic disorders caused by mutations in *EFHD2* are known, but unbiased studies using mass spectrometry, cDNA microarrays or RNA sequencing identified EFHD2 dysregulation in studies on brain tissue of human postmortem samples or mouse models of AD, Huntington’s disease, Parkinson’s disease and amyotrophic lateral sclerosis [4]. In addition, a potential role of EFHD2 in AD was suggested by its association with TAU in human AD brain tissue and by its possible self-aggregation [4].

In our recent study, we took a closer look at the neurogenic niche of the adult hippocampus of *Efhd2* knockout mice [7]. Using thymidine analogue incorporation assays, we showed that proliferation of hippocampal neural stem and progenitor cells was unchanged. In addition, survival of newborn neurons was markedly reduced in *Efhd2* knockout animals. This loss occurred already at the neuroblast stage, i.e. when newborn cells had committed towards the neuronal lineage.

Next, we focused on the dendritic morphology of newborn neurons, reflecting postsynaptic integration into the microenvironment of the adult molecular layer. There was a significant reduction of dendrite growth, dendrite complexity and spine formation in newborn *Efhd2* knockout neurons. As *Efhd2* deletion had positive effects on axonal growth and axonal transport *in vitro*, we hypothesized a cell-extrinsic effect of *Efhd2* on dendritic integration. This was confirmed by cell-specific deletion of *Efhd2* in adult newborn neurons by Cre recombinase.

Due to the links between EFHD2 and neurodegenerative diseases mentioned above, and due to the known decrease of adult neurogenesis in different mouse models of AD, we investigated the association of EFHD2 with TAU. We detected a profound increase of TAU and p-TAU in the hippocampus of adult *Efhd2* knockout mice. Thus, our data suggest that loss of EFHD2 leads to hippocampal tauopathy, which in turn impairs the integration of adult newborn neurons. Future studies will have to assess cause and relevance of increased TAU species in the hippocampus of *Efhd2* knockout animals. Strikingly, synaptic dysfunction and impaired adult neurogenesis are early features in mouse models of AD.

How could a cell-extrinsic effect contribute to increased intracellular TAU species in the *Efhd2* knockout hippocampus? Microarray analyses of the prefrontal cortex of *Efhd2* knockout mice revealed dysregulation of genes involved in axonal guidance, glutamatergic synapse formation, chemokine receptor signaling, focal adhesion formation and extracellular matrix receptor interaction, including guidance cues such as *Sema3c* (Semaphorin-3C) and *Efna3* (Ephrin-A3) [6]. Interestingly, the positive regulation of dendrite growth by Semaphorin-3C is mediated by CDK5 and both TAU and EFHD2 can be phosphorylated by CDK5 [4]. Hence, dysregulation of environmental factors regulating axon and synapse formation may mediate the cell-extrinsic effect of *Efhd2* deletion on adult hippocampal neurogenesis.
